# Fully automated segmentation and radiomics feature extraction of hypopharyngeal cancer on MRI using deep learning

**DOI:** 10.1007/s00330-023-09827-2

**Published:** 2023-06-20

**Authors:** Yu-Chun Lin, Gigin Lin, Sumit Pandey, Chih-Hua Yeh, Jiun-Jie Wang, Chien-Yu Lin, Tsung-Ying Ho, Sheung-Fat Ko, Shu-Hang Ng

**Affiliations:** 1 Department of Medical Imaging and Intervention, Chang Gung Memorial Hospital at Linkou, 5 Fuhsing St., Guishan, Taoyuan, 33382 Taiwan; 2 Department of Medical Imaging and Radiological Sciences, Chang Gung University, Taoyuan, Taiwan; 3 Clinical Metabolomics Core Laboratory, Chang Gung Memorial Hospital at Linkou, Taoyuan, Taiwan; 4 Department of Radiation Oncology, Chang Gung Memorial Hospital at Linkou and Chang Gung University, Taoyuan, Taiwan; 5 Department of Nuclear Medicine and Molecular Imaging Center, Chang Gung Memorial Hospital and Chang Gung University, Taoyuan, Taiwan; 6 Department of Radiology, Kaohsiung Chang Gung Memorial Hospital and Chang Gung University College of Medicine, Kaohsiung, Taiwan

**Keywords:** Magnetic resonance imaging, Deep learning, Hypopharyngeal cancer

## Abstract

**Objectives:**

To use convolutional neural network for fully automated segmentation and radiomics features extraction of hypopharyngeal cancer (HPC) tumor in MRI.

**Methods:**

MR images were collected from 222 HPC patients, among them 178 patients were used for training, and another 44 patients were recruited for testing. U-Net and DeepLab V3 + architectures were used for training the models. The model performance was evaluated using the dice similarity coefficient (DSC), Jaccard index, and average surface distance. The reliability of radiomics parameters of the tumor extracted by the models was assessed using intraclass correlation coefficient (ICC).

**Results:**

The predicted tumor volumes by DeepLab V3 + model and U-Net model were highly correlated with those delineated manually (*p* < 0.001). The DSC of DeepLab V3 + model was significantly higher than that of U-Net model (0.77 vs 0.75, *p* < 0.05), particularly in those small tumor volumes of < 10 cm^3^ (0.74 vs 0.70, *p* < 0.001). For radiomics extraction of the first-order features, both models exhibited high agreement (ICC: 0.71–0.91) with manual delineation. The radiomics extracted by DeepLab V3 + model had significantly higher ICCs than those extracted by U-Net model for 7 of 19 first-order features and for 8 of 17 shape-based features (*p* < 0.05).

**Conclusion:**

Both DeepLab V3 + and U-Net models produced reasonable results in automated segmentation and radiomic features extraction of HPC on MR images, whereas DeepLab V3 + had a better performance than U-Net.

**Clinical relevance statement:**

The deep learning model, DeepLab V3 + , exhibited promising performance in automated tumor segmentation and radiomics extraction for hypopharyngeal cancer on MRI. This approach holds great potential for enhancing the radiotherapy workflow and facilitating prediction of treatment outcomes.

**Key Points:**

• *DeepLab V3* + *and U-Net models produced reasonable results in automated segmentation and radiomic features extraction of HPC on MR images.*

• *DeepLab V3* + *model was more accurate than U-Net in automated segmentation, especially on small tumors.*

• *DeepLab V3* + *exhibited higher agreement for about half of the first-order and shape-based radiomics features than U-Net.*

**Supplementary information:**

The online version contains supplementary material available at 10.1007/s00330-023-09827-2.

## Introduction

Hypopharyngeal cancer (HPC) has the worst prognosis among all head and neck squamous cell cancers (HNSCC). The incidence and mortality of HPC continues to increase over the past decades [1]. Most patients with HPC have advanced cancer staging at presentation, and chemoradiation is currently considered as the mainstay of organ-preservation therapy. Previous studies have shown that magnetic resonance imaging (MRI) provides anatomic, qualitative, and quantitative functional information in clinical practice for staging, treatment planning, and response assessment of HNSCC [2–5].

Determining tumor contour is essential for radiation therapy planning, and MRI is widely applied for pretreatment delineation of tumor extension [6]. In addition, tumor contouring plays an essential role for radiomics analysis. Magnetic resonance (MR) radiomics can serve as a biomarker in the prediction of treatment outcomes [7], and survival in HNSCC [8]. However, manual contouring of tumors in a slice-by-slice fashion is labor intensive and prone to interobserver variations [9]. An effective tumor auto-segmentation tool is expected to improve the efficiency of the radiotherapy workflow.

In recent years, convolutional neural networks (CNNs) have been extensively used for semantic segmentation on medical image [10, 11]; however, its applications on HNSCC have been studied on computed tomography (CT) or PET/CT [12, 13], with the dice similarity coefficient (DSC) being 0.66 on CT and 0.74 on PET/CT, respectively. A few studies have focused on MRI; however, the accuracies of segmentation were rather low with the DSC ranging between 0.49 and 0.65 [14, 15].

U-Net, a CNN-based method for biomedical image segmentation, has been widely used recently [16–18]. However, one of the challenges of U-Net is the excessive downsizing by consecutive pooling layers, which makes it sensitive to the size of mask for training. To this end, DeepLab V3 + [19] architecture was introduced which utilized the atrous spatial pyramidal pooling (ASPP) to obtain multi-scale context information to improve the limit of consecutive pooling layers. DeepLab V3 + has been applied in many segmentation tasks, where the performances were reported to be superior to many state-of-the-art networks [20, 21].

The aim of this study was to evaluate the performance of DeepLab V3 + for automated segmentation and radiomics extraction of HPC primary tumors in MRI and compare with that of U-Net.

## Methods and materials

### Patients

This study retrospectively analyzed the dataset of patients with HPC stage T3 and T4 during 2006 to 2017. The Institutional Review Board approved the study, and informed consent was waved. The inclusion criteria were (a) newly biopsy-proven stage T3 and T4 (American Joint Committee on Cancer, AJCC) hypopharyngeal squamous cell carcinoma and (b) having complete our uniform chemoradiation scheme. The exclusion criteria were inaccessibility for follow-up, contraindications to MRI, history of previous head or neck cancers, second malignancies, renal failure, and mental health–related inability to cooperate.

We included 242 consecutive patients, of whom 12 were excluded because they had indistinct margins and 8 were excluded because their images had considerable artifacts. Accordingly, 222 patients were included in the final study sample, and their MR images were used as the dataset for analysis. We separated the dataset obtained from our 178 patients (80.2%) as a training dataset and those from the remaining 44 patients (19.8%) as a testing dataset. The training dataset was further divided into training and validation subsets by using fivefold cross-validation (executed using 35 or 36 images per fold). This step was implemented to ensure that the models’ performance would not be affected by data splitting, thus preventing overfitting.

### Treatment

All patients received intensity-modulated radiotherapy using 6-MV photon beams. The initial prophylactic field included gross tumor with at least 1-cm margins and neck lymphatics at risk for 46–56 Gy, then cone-down boost to the gross tumor area up to 72 Gy. Concurrent chemotherapy consisted of intravenous cisplatin, oral tegafur plus oral leucovorin.

### MRI data and image annotation

MRI data were acquired using three MRI scanners (Siemens, GE, and Philips). All patients underwent the standard MRI protocol in our institute. T1-weighted turbo spin echo (TSE) images (TR/TE: 400–760/11–16 ms) and T2-weighted fat-saturated (T2w-fs) TSE images (TR/TE: 4100–5300/80–92 ms) were acquired in axial and coronary planes. After an intravenous administration of gadolinium at a dose of 0.1 mmol/kg, a fat-saturated contrast-enhanced T1-weighted (cT1w-fs) TSE sequence (TR/TE: 400–680 ms) was obtained in the axial, sagittal, and coronal planes. The cT1w-fs images obtained in the axial plane were used for model training and testing, and the parameters were outlined as follows: field of view, 220 mm; slice thickness, 4 mm; acquisition matrix, 256 × 256 or 384 × 320; reconstruction matrix, 512 × 512; number of slices, 30–36; and scan time, 200–280 s.

Regions of interest (ROIs) capturing tumor contours throughout the primary tumor volume were segmented slice by slide on cT1w-fs MR images by two neuroradiologists (S.H.N., with 27 years of experience in head and neck imaging; C.H.Y., with 12 years of experience in head and neck imaging) via consensus, with the aid of T2-weighted MR images, using an in-house interface developed through MATLAB (Mathworks). Both neuroradiologists were blinded to the clinical outcomes. The labeled ROIs were used as the ground truth for the model training.

### Image preprocessing

Data augmentation was performed on each training image set such that the training model can be robust against the degree of enlargement, rotation, and parallel shift. Each image was randomly rotated within the range −10 to 10°. The range of the random shift was 0.9–1.1 of the image length and width. The image was zoomed by the range of 0.9–1.1.

### Network and training

**Fig. 1:**
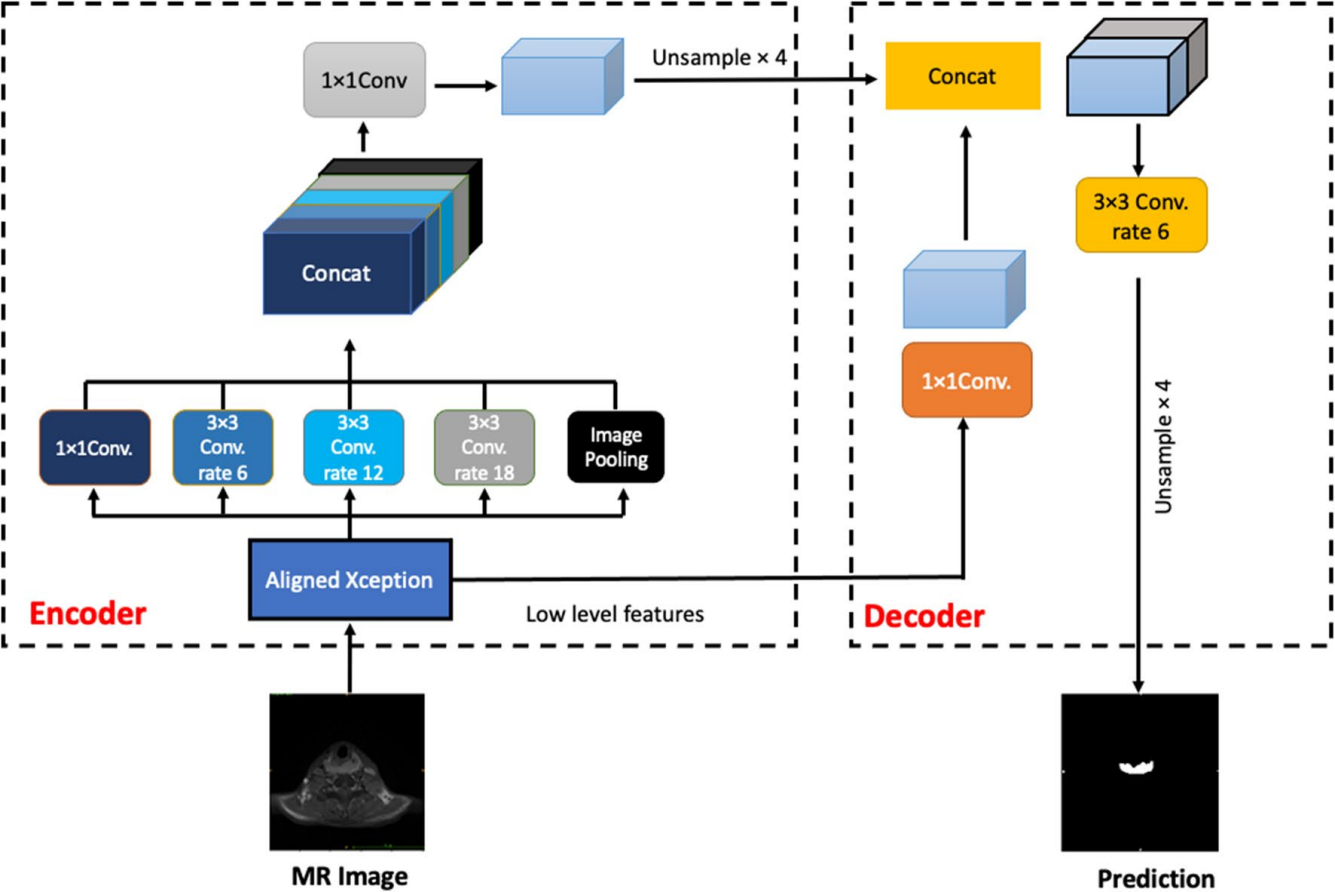
An illustration of the DeepLabV3 + . The encoder module encodes multi-scale contextual information by applying atrous (dilated) convolution at multiple scales, while the simple yet effective decoder module refines the segmentation results along object boundaries

Two types of network architectures were used for training and testing the performances of tumor segmentation: the U-Net as previous studies [18, 22], and DeepLab V3 + (Fig. 1), a decoder module with a depth separable convolution for ASPP layers to improve the object boundary detection [23]. The networks were trained with weight randomization and stochastic gradient descent Adam optimizer method [24]. The signal intensities of all images were normalized to a mean = 0 and standard deviation = 1 [25]. The learning rate was set to 0.001, and the number of epochs until convergence was 100 with batch sizes of 2. The network was trained using Keras 2.1.4 written in Python 3.5.4 and TensorFlow 1.5.0. The code for the DeepLab V3 + model is available at https://github.com/bonlime/ keras-deeplab-v3-plus.

### Performance evaluation

Each model’s segmentation performance was assessed using the following metrics [26]: (1) the DSC, a measure of spatial overlap calculated using the formula 2TP/(FP + 2TP + FN), where TP, FP, and FN represent true-positive, false-positive, and false-negative detections, respectively; (2) Jaccard index, calculated using the formula TP/(TP + FP + FN); and (3) average surface distance (ASD), calculated as the mean Euclidean distance between surface voxels in the segmented object and ground truth.

### Radiomics

To assess the reliability of predicted ROIs by the established models, we examined the radiomics features of tumor ROIs extracted by manual and automatic segmentation models. The radiomics features of tumors were calculated using Pyradiomics software [27] based on the 3D volumes of ROIs on the images (https://pyradiomics.readthedocs.io/en/latest/features.html). A total of 105 radiomics were extracted, which were divided into the following classes: first-order statistics (19 features), shape-based (17 features), gray-level co-occurrence matrix (GLCM, 24 features), gray-level run length matrix (GLRLM, 14 features), gray-level size zone matrix (GLSZM, 13 features), neighboring gray-tone difference matrix (NGTDM, 4 features), and gray-level dependence matrix (GLDM, 14 features).

### Statistics

Statistical analysis was performed using GraphPad Prism software version 8.0 for Mac. The two models’ performance metrics were compared using the Wilcoxon rank test. The differences in DSCs among the MRI scanners were assessed using the Kruskal–Wallis test. Bland–Altman plots were used to assess the agreement between the tumor volumes obtained from the automated models and those obtained by manual segmentation. Pearson’s correlation analysis was used to determine the correlations between the tumor volumes obtained from the automated models and those obtained by manual segmentation. The reliability of radiomics features of tumor ROIs was evaluated using intraclass correlation coefficient (ICC) obtained by manual and automatic segmentation models. The differences in DSCs with regard to tumor size and tumor histology were assessed using the Mann–Whitney *U* test.

## Results

### Patient characteristics

Table 1 presents the clinical and demographic features of the training (*n* = 178) and testing datasets (*n* = 44). The median patient age was 53 years (range: 33–83 years). No statistically significant differences were found regarding the clinical or demographic characteristics between the training and testing datasets.

**Table 1:** Patient demographics

Variable	Training	Testing	*p* value
Patient number (*n*)	178 (80.1%)	44 (19.8%)	
Age (year)	53.6 ± 9.59	52.2 ± 10.1	0.39
Differentiation grade			0.58
Well/moderate	84 (47.2%)	26 (50%)	
Poorly	21 (11.8%)	4 (9.1%)	
Anaplastic	74 (41.6%)	14 (36.4%)	
T stage			0.86
3	43 (24.2%)	9 (22.7%)	
4	135 (75.8%)	35 (77.3%)	
N stage			0.57
N0	31 (17.4%)	5 (11.4%)	
N1	18 (10.1%)	6 (13.6%)	
N2	102 (57.3%)	24 (54.5%)	
N3	27 (15.2%)	8 (18.2%)	
M stage			0.93
0	169 (94.9%)	42 (95.5%)	
1	9 (5.1%)	2 (4.5%)	

### Model performance

**Fig. 2:**
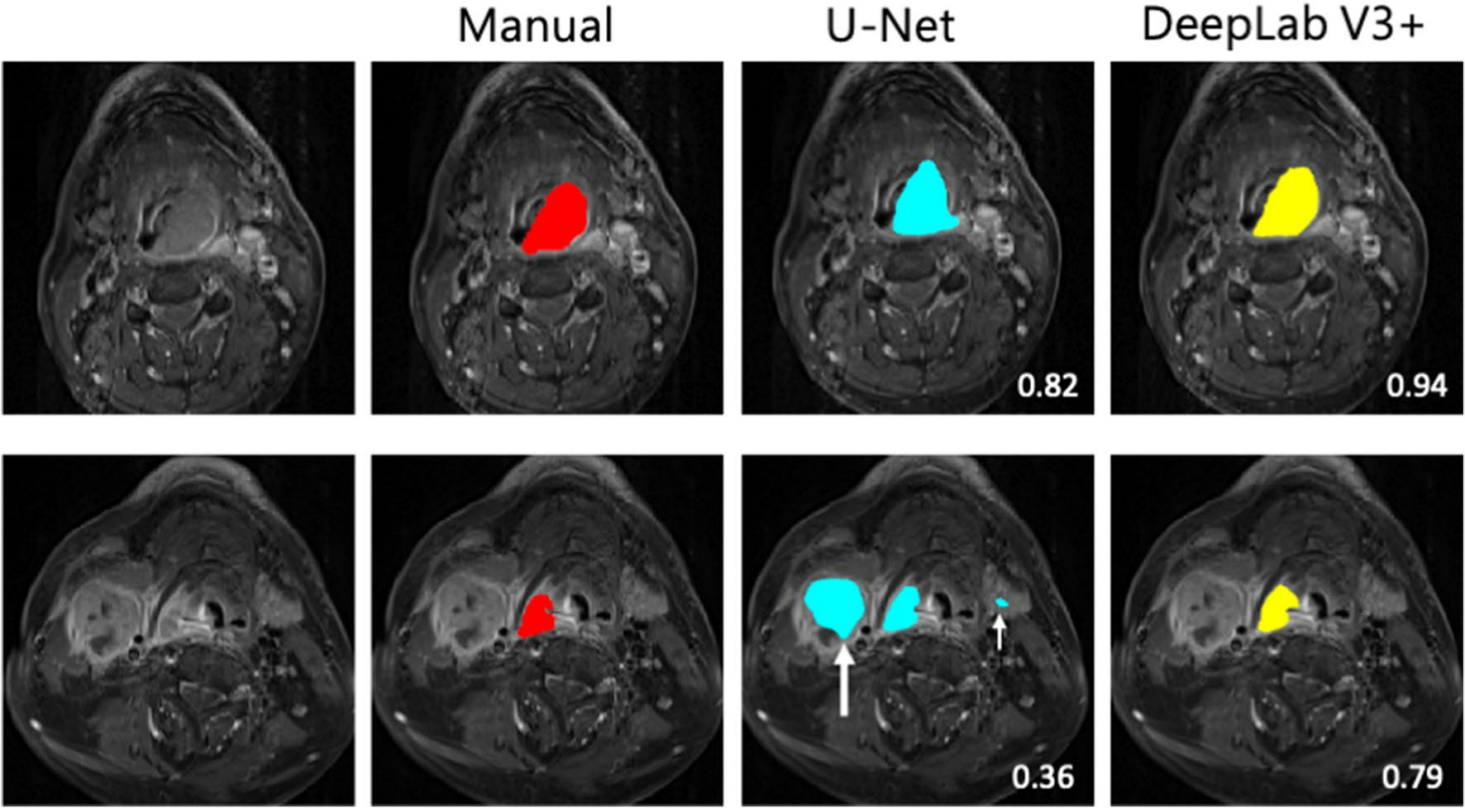
Examples of tumor segmentation in two patients with a large hypopharyngeal tumor (upper row) and a small hypopharyngeal tumor (lower row) on cT1w-fs images. The tumors are delineated in red for manual segmentation, in blue by U-Net model, and in yellow by DeepLab V3 + . The numbers indicate the DSC scores. The white arrows indicate the false positives in the predicted segmentation. The large white arrow points to a large metastatic lymph node, while the small white arrow points to left submandibular gland

Table 2 presents a summary of the DeepLab V3 + and U-Net models’ performance. DeepLab V3 + significantly outperformed U-Net in all metrics. DeepLab V3 + had a higher DSC (*p* < 0.05) and Jaccard index (*p* < 0.001) but a lower ASD (*p* < 0.001) than did U-Net. The models’ performance levels were similar across the images obtained from the 3 different MRI scanners employed in this study (*p* = 0.78 and 0.83 for U-Net and DeepLab V3 + models, respectively) (Supplementary Table 1). Representative figures (Fig. 2) show delineation of primary tumors in a large HPC and a small HPC using U-Net and DeepLab V3 + models respectively. After analyzing the 421 image slices in the testing dataset, we found that U-Net yielded 41 (9.7%) FPs, which occurred most frequently in the adjacent lymph nodes (*n* = 30), followed by the submandibular gland (*n* = 14) and the tongue base (*n* = 1). DeepLab V3 + yielded only 2 (0.5%) FPs in the submandibular gland.

**Table 2:** Segmentation performance results of U-Net and DeepLab V3 + models

Evaluation metrics	U-Net	DeepLab V3 +	*p*-value
DSC	0.75 (0.69, 0.77)	0.77 (0.74, 0.79)	< 0.05
Jaccard index	0.62 (0.59, 0.65)	0.67 (0.64, 0.69)	< 0.001
ASD^*^	1.89 (1.77, 2.03)	1.23 (1.12, 1.34)	< 0.001

Data are presented as medians with 95% confidence intervals. *DSC*, dice similarity coefficient; *ASD*, average surface distance. ^*^Units in millimeters

### Impact of tumor volume to prediction accuracy

**Fig. 3:**
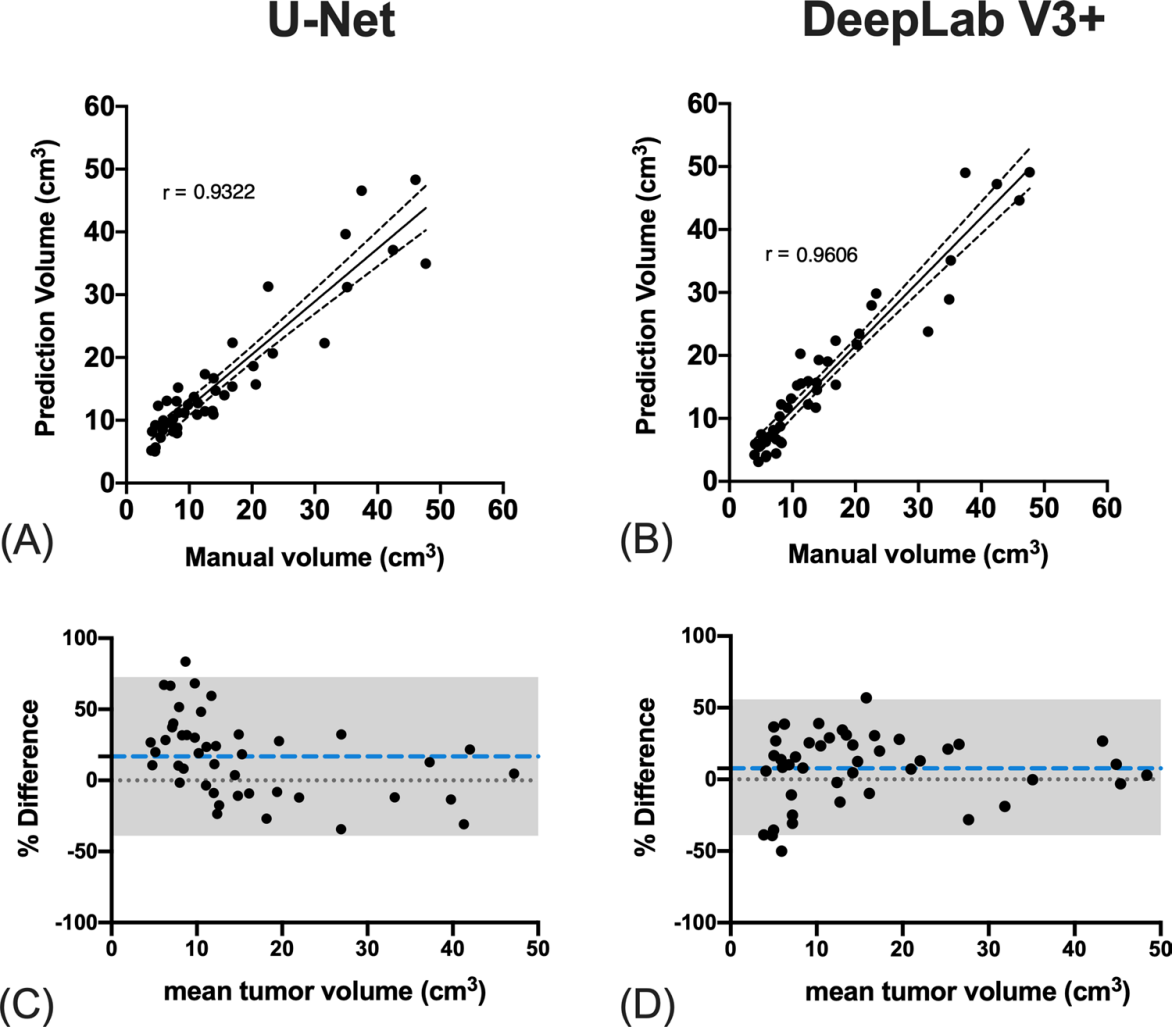
**A**, **B** Correlations of tumor volumes between the manual and predicted tumor volumes using DeepLab and U-Net models. **C**, **D** Blade-Altman plots of the tumor volumes between the manual and the model-derived volumes. The percentage difference was calculated by the difference of model-derived volume minus the mean of the two measurements. The blue dot line indicated the bias of difference. The gray shade area indicated the 95% limits of agreement

Figure 3 shows that the predicted tumor volumes by U-Net and DeepLab V3 + models were highly correlated with those delineated manually (*r* = 0.93 and 0.96 for U-Net and DeepLab V3 + models, respectively, *p* < 0.001 for both). The Bland–Altman plots demonstrated that the U-Net model had a higher mean difference bias compared to the DeepLab V3 + model (bias:16.6% vs 7.69%; 95% limit of agreement: −30 to 72.6% vs −40.4 to 50.7% for U-Net and DeepLab V3 + models, respectively).

**Fig. 4:**
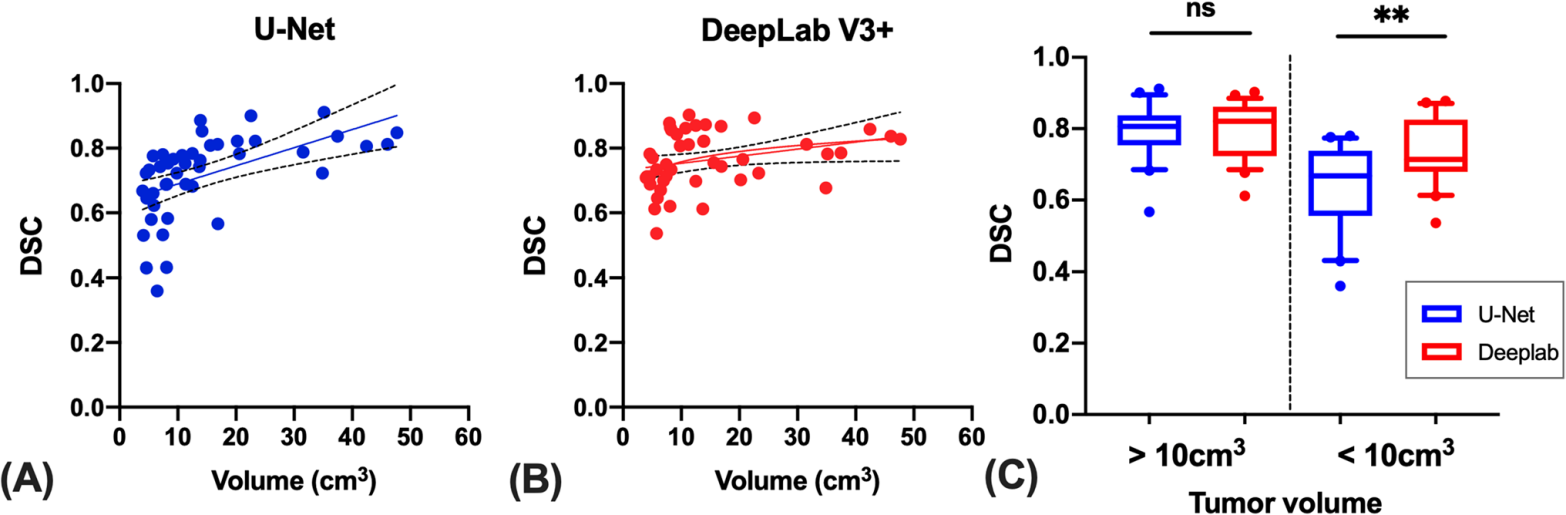
**A**, **B** Scatter plots of predicted tumor volumes versus DSC using U-Net and DeepLab models. **C** Comparisons between the DSCs of the models in small and large tumors. ns, not significant; **p < 0.001

Figure 4 plots the relations of DSC of U-Net and DeepLab V3 + models to the tumor volume of ground truth. The U-Net model showed lower DSC than DeepLab V3 + in small tumors. Through a stepwise approach, we subdivided our dataset into subgroups by using various thresholds and then compared the performance of these thresholds in producing differences in DSCs between tumors. We observed that a threshold of 10 cm^3^ produced the most pronounced differences in DSCs between large and small tumors. We conducted a subgroup analysis by dividing the test dataset into two subgroups according to tumor volume: small (volume < 10 cm^3^; *n* = 21) and large (volume > 10 cm^3^; *n* = 23) groups. In the small group, DeepLab V3 + had significantly a higher DSC (0.74, 95% CI: 0.72–0.76) than did U-Net (0.70, 95% CI: 0.68–0.72; *p* < 0.001); however, in the large group, the models did not differ significantly in DSCs (DeepLab V3 + : 0.8, 95% CI: 0.78–0.82; U-Net: 0.79, 95% CI: 0.76–0.82; *p* = 0.72; Fig. 4C).

### Reproducibility of radiomics features

**Fig. 5:**
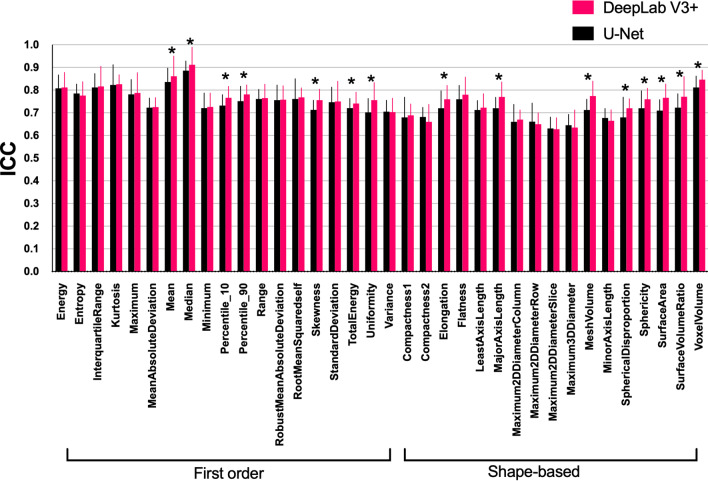
Intraclass correlation coefficients (ICC) of radiomics features of first-order and shape-based features between manual and model predicted ROIs using U-Net and DeepLab models

Figure 5 shows the ICC values of radiomics features extracted by manual and predicted ROIs using U-Net and DeepLab V3 + models. For the first-order features, both models exhibited high agreement (ICC range: 0.71–0.89 for U-Net model and 0.71–0.91 for DeepLab V3 + model). The DeepLab V3 + model had significantly higher ICCs for the following 7 of the 19 features: mean, median, 10 percentile, 90 percentile, skewness, total energy, uniformity (*p* < 0.05). For the shape-based features, both models exhibited moderate to good agreement (ICC range: 0.63–0.81 for U-Net model and 0.63–0.85 for DeepLab V3 + model). The DeepLab V3 + model had significantly higher ICCs for the following 8 of the 17 features: elongation, major axis length, mesh volume, spherical disproportion, sphericity, surface area, surface volume ratio, voxel volume (*p* < 0.05). The ICCs of the other radiomics features were moderate or low for both models (ICC range, 0.41 to 0.71 vs 0.43 to 0.73 for U-Net vs DeepLab V3 + model, respectively.)

## Discussion

The use of deep learning for assessing head and neck cancer is an evolving field with considerable scientific and clinical potential. However, only a few studies have investigated the use of deep learning models for specific types of head and neck cancer on MR images. Our study used CNN-based models, namely DeepLab V3 + and U-Net, for automatic segmentation and radiomic features extraction of HPC on MR images. DeepLab V3 + exhibited superior segmentation performance to U-Net and other CNN models [14, 15], providing a promising model for improving the accuracy of head and neck cancer auto-segmentation using AI networks. We also demonstrated the generalizability of radiomic features derived by the models, which can benefit future research. We recommend the use of modern MRI scanners in facilities that handle high volumes of hypopharyngeal cancer patients. These scanners offer superior soft tissue contrast, as well as strong performance potential to support DL models in automated tumor segmentation and radiomics feature extraction.

We used a cT1w-fs sequence for model training because it clearly depicts tumor boundaries and is preferred for tumor extent identification in clinical practice. A T2w-fs sequence is also valuable for lesion extent evaluation; hence, we draw ROI on cT1w-fs images with the aid of T2-fs images in this study. Wong et al reported that U-Net exhibited similar performance levels in segmenting nasopharyngeal carcinoma tumors on T2w-fs and cT1-fs images; accordingly, T2w-fs images may be suitable for automatic tumor segmentation in clinical scenarios that require avoiding contrast agents [28].

Manually tumor delineation in radiotherapy planning is time-consuming and variable based on the level of expertise. While auto-segmentation algorithms may potentially save effort and time of a radiotherapist, the challenges in accurate tumor definition remain. Bielak et al [14] investigated the HNSCCs using a 3D DeepMedic CNN architecture with 7 MRI channel input and scored the average DSC of 0.65. Schouten et al [15] performed multi-view CNN for various HNSCCs, and presented an average DSC of 0.49 for all cases and 0.57 for HPC. In the current study, we specifically trained on HPC patients with U-Net and DeepLab V3 + models, and achieved DSCs of 0.72 and 0.77, respectively. The results suggest that training a dedicated CNN model with U-Net or DeepLab V3 + for HPC would attain better prediction of the target tumor contour, and may offer the potential improvement of artificial intelligence (AI)–based auto-segmentation.

The accuracies of segmentation of CNN have been reported to be positively related with tumor size [14, 15, 18]. Schouten et al [15] reported that multi-view CNN produced improved segmentation results with larger tumor volumes, and tended to overestimate the tumor volume with FPs around the primary tumor or in adjacent enlarged lymph nodes. In our study, U-Net had significantly lower DSCs than DeepLab V3 + , particularly for small-size tumors. A reason could be attributed that U-Net utilized the general fully convolutional network, which is sensitive to the mask size of input data due to excessive downsizing by consecutive pooling layers [29], while DeepLab network with ASPP can capture context information at multiple spatial scales and produce accurate segmentation on input images with different size. Our Bland–Altman plots revealed that U-Net had a higher mean difference bias than did DeepLab V3 + (16.6% vs 7.69%), probably because U-Net had more FPs in the adjacent lymph nodes and submandibular gland. In radiotherapy, FP findings can lead to erroneous treatment plans with undesirable toxicity. The highest tolerable error for tumor segmentation in radiotherapy may vary depending on the patient’s medical status, tumor type, location, and prescribed radiation dose, and should be kept as low as possible. Although U-net has been showing potential in segmenting medial images, radiotherapy physicians should recognize that it may yield TPs near the primary lesions, as shown in our cases and as reported by studies applying it to segmentation of the knee meniscus [30] and lung nodules [31].

Our results demonstrated that the radiomics parameters of HPC extracted by both U-Net and DeepLab V3 + models were robust for the first-order and the shape-based features. This finding suggests that such CNN-based automated segmentation approach could improve the high-throughput extraction of radiomics features in a timely manner. Xue et al [32] showed that the first-order radiomics features had significantly higher ICCs than the other texture features. Our study proved that the first-order features remained reliable using the CNN-based approach with U-Net or DeepLab V3 + model, in line with high reproducibility of first-order radiomics features of diffusion-weighted imaging for cervical cancer using U-Net [18]. In addition, our results also demonstrated that DeepLab V3 + exhibited shape-based features with higher reproducibility than U-Net. Since the shape features depend solely on the segmentation masks related to the accuracy of tumor contours, this finding suggests that DeepLab V3 + would be a feasible extraction method for MRI radiomics features that would potentially help reliable outcome prediction of HPC specifically.

Despite the abovementioned strengths, our study had some limitations. First, this study demonstrated that DeepLab V3 + can accurately segment HPC on MR images. However, further research is necessary to define the clinical acceptability of the model and estimate how much time it could save in the radiotherapy workflow. Second, our study showed that DeepLab V3 + extracted MRI radiomics features of HPC with good reproducibility, but to what extent such extracted feature aid reliable outcome prediction would be the subject of further investigation. Third, the findings of a single-center study may not be generalizable to other hospitals. Although our sample size of 220 patients was relatively small, a total of 2085 tumor slices were used. Nonetheless, larger multicenter studies are warranted for external validation and prospective testing. Our results suggest that the trained models exhibited similar performance levels when tested on images from three different MRI scanners. Therefore, the models may be applicable to images from MRI scanners with similar imaging parameters in other hospitals.

In conclusion, our study revealed that both DeepLab V3 + and U-Net models produced reasonable results in automated segmentation and radiomic features extraction of HPC on MR images, whereas DeepLab V3 + had a better performance than U-Net.

## Supplementary Information

Below is the link to the electronic supplementary material.

Supplementary file1 (PDF 30 kb)

## Abbreviations

ASPP: Atrous spatial pyramid pooling
CNN: Convolutional neural network
CT: Computed tomography
cT1w-fs: Fat-saturated contrast-enhanced T1-weighted
DSC: Dice similarity coefficient
HNSCC: Head and neck squamous cell cancers
HPC: Hypopharyngeal cancer
ICC: Intraclass correlation coefficients
MRI: Magnetic resonance imaging
PET: Positron emission tomography
ROI: Region of interest
T2w-fs: Fat-saturated T2 weighted

## Declarations

### Guarantor

The scientific guarantor of this publication is Shu-Hang Ng.

### Statistics and biometry

No complex statistical methods were necessary for this paper.

### Study subjects or cohorts overlap

None of the study subjects or cohorts has been previously reported.

### Methodology

• retrospective

• diagnostic or prognostic study

• performed at one institution
