# Supplementary material for: Fully automated segmentation and radiomics feature extraction of hypopharyngeal cancer on MRI using deep learning
Source: Eur Radiol. 2023 Jun 20;33(9):6548–56. doi: 10.1007/s00330-023-09827-2 (PMC10415433; doi:10.1007/s00330-023-09827-2)
Supplement: Supplementary file 1 — Supplementary file1 (PDF 30 kb) [file 330_2023_9827_MOESM1_ESM.pdf]

Fully Automated Segmentation and Radiomic FeatureExtraction of Hypopharyngeal Cancer on MRI Using Deep Learning

ELECTRONIC SUPPLEMENTARY MATERIAL

Supplementary Table 1

Segmentation accuracies of trained models across different MRI scanners

Vendor		Siemens	GE	Philips	
Model name		TRIO TIM	750	Ingenia	**p**-value
Number of Patient		21	11	12	
DSC	U-Net	0.76 (0.71, 0.79)	0.75 (0.70, 0.77)	0.74 (0.68, 0.78)	0.78
	DeepLab V3+	0.78 (0.73, 0.81)	0.76 (0.72, 0.79)	0.77 (0.73, 0.80)	0.83

Data are presented as medians with 95% confidence intervals in parentheses.
